# Am I my students’ nurse? Reflections on the nursing ethics of nursing education

**DOI:** 10.1177/09697330231193858

**Published:** 2023-09-28

**Authors:** Paul Snelling

**Affiliations:** 8709 University of Worcester, UK

**Keywords:** Code, cultivation of character, nursing ethics, nursing education, analogy, student nurses

## Abstract

Despite having worked in higher education for over twenty years, I am still, first and foremost, a practicing nurse. My employer requires me to be a nurse and my regulator regards what I do as nursing. My practice is regulated by the Code and informed by nursing ethics. If I am nurse, practicing nursing, does that mean that my students are my patients? This paper considers how the relationship that I have with my students can be informed by the ethics of the nurse/patient relationship. After some initial theoretical preparation concerning argument from analogy, the paper identifies some areas for comparison between the two relationships. Areas of similarity and difference identify two areas of concern: Nurse education and educators regularly engage in coercion and surveillance in an attempt to increase student success, both of which would be considered outside nursing ethics. It is concluded that these coercive practices are not conducive to an environment where character is cultivated. Despite current financial and workforce pressures, nurse lecturers and more especially their managers would do well to return to the professional ethics of nursing to question and guide their practice.

## Introduction

Despite being employed full time within higher education for over 20 years, I proudly remain, first and foremost, a nurse. During lectures I am sometimes asked if I am a ‘still’ a nurse, or whether I do any ‘real’ nursing, or whether I still see any patients, or if I miss nursing. In response, and to illustrate the working of the register, I log on to the Nursing and Midwifery Council (NMC) website, search for my name, and after an anxious pause, record of my registration is displayed on the screen. I’ll admit that I would be a little nervous returning to a ward for a shift, but I could do it, and in class this leads to a discussion about one of the fundamental tenets of being a professional, of understanding our scope of practice and competence and practicing within it, undertaking self-directed learning to address self-identified needs. Being a registered nurse was an essential requirement for my employment as lecturer in nursing. If I were to forget to re-register or was suspended or removed from the register I could, probably would, be dismissed, even if my teaching was restricted to general material like anatomy or research methods.

In the UK, nursing students in placement must be assessed by practice and academic assessors who are registered nurses.^1^ However, teaching and assessment for the ‘theory’ content does not require me to be a registered nurse, only that I am ‘appropriately qualified and experienced’ (p.11).^2^ When I renewed my registration, the hours requirement was satisfied by my employment as a teacher and researcher, and my five required pieces of reflection which must be related to the Code,^3^ concerned my professional practice of teaching and research. My practice related feedback came from student teaching evaluations and personal testimony. The NMC is very clear that my work is covered by the Code:^3^

Nurses, midwives and nursing associates must act in line with the Code, whether they are providing direct care to individuals, groups or communities or bringing their professional knowledge to bear on nursing and midwifery practice in other roles, such as leadership, education, or research. The values and principles set out in the Code can be applied in a range of different practice settings, but they are not negotiable or discretionary (p.3).

My personal conduct outside my work is also subject to the Code: My social media posts are ‘responsible’^4^ and I most certainly would offer emergency assistance if I were able to and considered I was competent. NMC Standards require that my university ensure that I act as ‘a professional role model at all times’ (p.11).^2^ Both my employer and my regulator regard me as a nurse and what I do as nursing.

The rhetorical question in the title of this paper concerns the implications of this observation. Working as a nurse and being liable to be judged according to the precepts of the Code raises the important question of the nature of the relationship that I have with students in general and with individual students. Are these relationships built, maintained, and terminated within the bounds of the patient–nurse relationship, regulated through the Code and informed by nursing ethics? Should they be? And if they are, what can an examination of the relationship between nurse and patient tell me about the relationship between me and my students? Much of what follows concerns my practice as a nurse and an academic, but many of the points also relate to staff/student relationships in higher education more generally.

The paper proceeds in three sections. Since this is a work of personal philosophical reflection, I need make my theoretical basis clear. The regulatory framework has already been introduced, but this alone is insufficient, so the first section sets out very briefly my understanding of and approach to nursing ethics, augmented by some explanatory detail about argumentation from analogy. The second section introduces some areas where comparison between the nurse–patient and teacher–student relationships is of interest. Each of these areas stands in need of deeper engagement but taken together they articulate two relationships which share much common ground but with some important differences. The final section considers whether the differences identified in practice are justified and considers some implications particularly regarding the cultivation of character.

## Nursing ethics and the nature of analogical argument

I have recently contributed an entry on nursing to an encyclopaedia of applied ethics,^5^ and in it I placed nursing ethics within two larger fields: professional ethics and healthcare ethics. The material on professional ethics argued that ethical decision making is inevitably undertaken in the context of codes of ethics, exemplified by the introductory section of this paper. The material on nursing ethics as healthcare ethics was explained in the draft abstract like this:

Nursing ethics can also be considered as a subset of healthcare ethics. Historically this field has been dominated by medicine (medical ethics), but since the professionalisation of nursing and other professions such as physiotherapy, distinct ethical claims for nursing have been articulated. Nurses and their codes articulate care rather than treatment often via the concept of compassion, less visible in other healthcare professions. Compassion is demonstrated not solely in actions, but in persons, and agent centred accounts of ethics, from the virtues to feminist ethics and to relational care ethics find resonance with the moral claims made by nurses and for nursing. Ultimately, nursing ethics is rooted in relationships.^5^

Though I am not a committed virtue ethicist, I have come to understand that competing moral theories in traditional accounts, Kantian deontology and Millian consequentialism, cannot offer a full account for nursing ethics. Duty-based and consequence-based theories both require moral agents to have a think about available options for action, and assess them dispassionately, by considering a universal law, or by calculating likely consequences. Although each theory comes with nuances and in myriad varieties, this need for detached thinking to the exclusion of emotional engagement within relationships provides, for many, a fatal objection to any unitary theory and a strong reason to adopt ‘pragmatic pluralism’.^6^ Some degree of agent-centred moral analysis is inevitable, as is consideration about how people – students – become moral agents. The cultivation of character is of interest to all.

Thinking by comparison or analogy is a common process, well known in bioethics^7,8^ with variations used by nurses in practice. When we compare situations (patient care, perhaps), seeking points of similarity and difference, or compare our situations with others and draw conclusions, we begin to engage with argumentation from analogy. Guiding decisions by precedent is familiar to lawyers, but also within educational practice: Student A was treated like this, the case of student B is similar so must be treated the same unless there are relevant differences, an application of a minimal requirement for justice attributed to Aristotle: Equals must be treated equally, and unequals must be treated unequally.^9^ More formally, the process of argumentation from analogy proceeds like this:^10^

S is similar to T in certain (known) respects.

S has some further feature Q.

Therefore, T also has the feature Q, or some feature Q* similar to Q.

For this paper the structure is amended slightly to emphasise normative rather than factual elements

S is similar to T in certain (known) respects.

S has some further normative feature Q.

Therefore, T also ought to have the feature Q, or some feature Q* similar to Q.

S is the relationship between nurse and patient. T is the relationship between nurse academic and student. Q is (collectively) the normative elements of ‘nursing ethics’, directed in part by the Code. This could be caveated and explained further, but the aim of this paper is not to offer a formal proof for any normative position, but simply to illuminate some factual features of the pair of relationships, and to extend those comparisons to regulatory and normative features. The direction of travel will be towards a conclusion which argues that in important aspects, educational institutions and the people who work within them fail to treat their students like they should, and if this is a conclusion which does not necessarily require argumentative steps from analogy, the comparative process nevertheless illuminates reasoning in important ways.

A key feature of the argument is that in both sets of relationships one of the individuals is a registered nurse and working as a nurse. But not all university teachers are nurses and not all teachers of nurses are themselves nurses. How is the comparison and the relationship different if the lecturer is not themself a nurse? And how does the relationship between nurse lecturer and employing organisation bear comparison with professional nursing practice within employment, informed by evidence and governed, allegedly, by the notion of *individual* accountability? The next section simply points to some areas of comparison, some factual and obvious, some normative and contestable, summarised in Table 1.

**Table 1.** Points of comparison.

Nurse academic/student nurse	Nurse / patient
Registered as nurse	Registered as nurse
Practicing as a nurse	Practicing as a nurse
Practice covered by code	Practice covered by code
Confidentiality and disclosure	Confidentiality and disclosure
Close relationships	Close relationships
A power dynamic is present and maintained	A power dynamic is present but minimised
Student centred learning	Patient centred care
Personal autonomy of student assumed (but…)	Personal autonomy of patient assumed (but…)
Obligation of student	Obligation of patient
A relationship based on care	A relationship based on care

The first three points in the table, registration and practice as a nurse within a code have been addressed. Given the weight of the documentary statements cited, these points of comparison need no further explanation. There are no codes of ethics or practice for university lecturers in the UK that I am aware of, certainly none that function in the same way as the quasi-legal NMC Code. There is no professional regulator for university lecturers. I am bound by policies in the course of my employment, as are most nurses, and yet we are members of a profession built upon the notion of professional autonomy. The comparison starts with two examples when educational practice might differ depending on whether the lecturer is a registered nurse or not.

## Confidentiality and disclosure

What if a student nurse tells me something in the course of an interaction that I believe I cannot keep confidential? The rules for disclosure of confidential information within the nurse/patient relationship are quite clear albeit guidance from regulators, including the NMC is very poor.^11^ Since I practice as a nurse during my work in education, I would be similarly bound if a student disclosed poor practice on placement if it could lead to serious patient harm, but if a student disclosed something about themselves, unrelated to practice, then guidance for me as a nurse and a university colleague who is not a nurse appears to be different, especially in relation to the possibility of harm to the student or others. The risk of harm to student alone appears to justify disclosure according to some university policies^12,13^ but not for health professionals.^11^ The threshold for disclosure for me working as a nurse appears to be higher than for a non-nursing colleague. This may be important for a student disclosing personal information.

## Close relationships with patients/students

The nature of any relationship between student and lecturer is different in its possibilities depending on whether the student is regarded as a patient where the relationship is bound by the Code which requires that nurses must: ‘stay objective and have clear professional boundaries at all times with people in your care (including those who have been in your care in the past), their families and carers’ (p.21).^3^ This is not a paper about the deficiencies – and there are many – of the Code^14^ but the wording is nevertheless of relevance if we are seriously to look to the document for guidance. If the intention of the paragraph is to prevent nurses exploiting their position by becoming over familiar or intimate with patients, it is devoid of the nuance contained in guidance produced by the Professional Standards Authority^15^ and the General Medical Council^16^ which are more trusting of relationships between professional and patient, requiring openness and accountability rather than outright prohibition, especially in regard to former patients.

Thankfully, universities have come a long way since the times of predatory lecturers satirised in Malclom Bradbury’s novel *The History Man.*^17^ However, recent concerns about university culture resulted in guidance from Universities UK that recommended that ‘close, personal relationships between staff and students are strongly discouraged by universities’ (p.35),^18^ and this nomenclature can be seen in university policies,^19^ stronger in some places than others. My own university ‘prohibits’^20^ intimate and sexual relationships between staff and students, but in rightfully seeking to prohibit misconduct and exploitation, the policy appears to exclude, unjustly, the possibility of genuine and lasting relationships – sexual or otherwise – as a result of an oversimplistic approach similar to that of the NMC.^21–23^ There is no mention in any university policy that I am aware of that discourages or attempts to prohibit relationships with former students.

## A power dynamic is present

The reason that health professionals are not allowed to enter personal relationships with their patients is the asymmetrical nature of the relationship and the inherent power dynamics and the subsequent potential of abuse. In nursing education, deference is dispatched to a bygone age replaced by informal clothes and first name terms, and yet power remains visible in some of the policies and practices that I shall return to. Curricular content is decided by the NMC, implemented by universities. Students generally have few opportunities for genuine choice about patterns of learning and assessment, and their practices and habits are subjected to all manner of officially sanctioned disapproval and censure. For example, university attendance policies ooze power and surveillance. From City University: ‘You are expected to attend all of your scheduled timetable. Attendance and engagement is monitored from the day you begin your programme.’^24^ I return to attendance policies below; they are a clear manifestation of the power dynamic between university – and by extension to its academic staff – and students, but other examples can be identified. I can require my students to attend a meeting, require them to explain their conduct, monitor their whereabouts and activity and regard their study habits and performance as fitting subjects for disapproval and blame, all part of normal practice for many within universities, maintained and promoted. Most obviously, I can fail work and prevent progression and completion, and the university is required to sign a declaration of health and character for all student nurses,^25^ without which they cannot register. For the corresponding feature of the nurse/patient relationship, the power imbalance persists, albeit ameliorated by consent and injunctions that it be recognised and minimised as part of patient centred care. The important distinction is that in nurse/patient relationship there is a clear obligation to recognise and minimise power whereas in the student/teacher relationship power is accepted and utilised.

## Patient centred care / student centred learning

When I meet a student or students, activities are based principally on their interests and for their benefit rather than mine. It’s not a reciprocal arrangement; the purpose of the transaction (if I can call it that) is to enhance student learning. There are some exceptions and limits, for example, when I investigate a complaint or sit on a fitness-to-practice panel, but these are unusual, and have me acting in a specific role, for example as Head of Department. If I were to pass an assignment that did not meet learning outcomes or fail to report a clear case of academic misconduct, though this might be considered in the student’s individual interest it would not serve the interests of the profession or society. Something similar could be said about a clinical nurse whose activity benefits patients within the limits of the law and professional practice, who would be equally censured if acting for her patient comprised others.

The ‘philosophy’ of patient centred care is central to nursing, regarded as axiomatic in codes and practice worldwide. There is also an analogous literature on student centred learning,^26^ not restricted to nursing departments. NMC standards for pre-registration nursing programmes do not perhaps go quite as far as some of the literature on student centred learning. Programmes are tightly controlled, but there is a whole section in the Standards on ‘student empowerment’ including ensuring that students ‘are supervised according to their individual learning needs, proficiency, and confidence (p.9).^2^ Whether this section meets the necessary features of student-centred learning is a discussion for another day. However, it is noteworthy that many of the empirical papers in Berg and Lepp’s^25^ review regard student centred learning as an intervention used to increase other goods, such as student knowledge or abilities or self-reliance. It is of instrumental value, and while this instrumentality is also seen in literature on patient centredness^27^ and the moral principle of respect for autonomy perhaps most visible within rule consequentialist^28^ accounts, there is also a clear moral imperative for patient centredness, but apparently not student centredness, for its own sake.

## Personal autonomy is assumed (but illusory)

I am fond of saying to my students that I am not their boss. You are an adult, I say, an autonomous learner, and it’s up to what you do, how you study. Only you can decide between competing values and demands on your time. The term ‘adult learner’ which relies on personal autonomy, is both descriptive and evaluative. To say that a person is an adult learner is to say, superficially, that they are both adult and a learner: over 18^1^ and registered on an educational programme. Evaluatively the term is loaded with assumptions about how an adult leaner accepts responsibility and ought to behave. Personal autonomy is also assumed within healthcare minimally by the requirement for consent and the bounds of mental capacity legislation and processes, recognised as central to healthcare ethics, albeit open to both manipulation through nudges^29^ and outright opposition.^30^ In this special issue Kristjansson and Thorarinsdottir^31^ discuss the fascinating concept of ‘constrained participation’ and its subconcepts ‘fought-for-participation’ and ‘forced-to-participation’ in relation to patient care, but I was struck on reading the paper how these ideas, particularly the latter, translate to educational practice. I was reminded how oftentimes students’ shoulders slump at the prospect of participatory activity and how in an echo of ‘paternalistic anti-paternalism’ I can virtually demand participation in a manner of my choosing whilst simultaneously claiming student centredness and the type of choice that accompanies authentic autonomy.

## Obligations of learner and patient

As Gerald Dworkin^32^ notes, any moral account that values autonomy has a problem with obligations. In the two sets of relationships, it is relatively easy to identify obligations on the part of lecturer and nurse. But many argue that there are also obligations on the part of student^33^ and patient. These may follow the form of the hypothetical rather than the categorical imperative: ‘If you want to feel better you should stop smoking.’ ‘If you want better marks you should study harder’. But there are also plenty who would extend these obligations to a general duty to maximise physical and mental health through healthy behaviour^34^ or to study as hard as able to get the best marks possible.

The nature of these sets of obligations can both be, to some extent, other-regarding. Other people have a legitimate interest in my health, especially those who love me and share my life and while this is not determinative it is worthy of consideration.^35^ It is also argued that scarcity of resources means that there is an obligation to be healthy to avoid drawing from the general healthcare pool, and for some, failure to meet these obligations should have consequences in terms of resource allocation.^36^ Other-regarding arguments for student obligations are weaker, but can be made for nursing and healthcare students who have patient facing obligations. A better pass grade makes a better nurse, or so it could be argued. How then, do we regard students who do just-enough-to-pass and have no desire to do better because they are lacking in innate ability, or a supportive environment, or who have other responsibilities, or who are simply poorly motivated? If there can be patient/student obligations which they fail to meet, can they be blamed, and by whom? There is a literature on the wrongness of victim blaming^37^ but blaming students for failure to meet ‘expectations’ set out by universities in documents like the one from City University^24^ cited earlier is less examined, similarly unjustified and probably as common. The NHS sets out patients’ responsibilities in the NHS constitution^38^ but the language is emollient, presented as a request rather than a statement of obligation or expectation (Please keep appointments, or cancel within reasonable time).

## A relationship based on care?

Care is axiomatic in nursing. Much of the literature on caring in teaching concerns compulsory education, but there is a literature on care in HE^39^ and qualitative research with academics and students highlights its features for those involved. Perhaps care is expressed most meaningfully in the relationships that develop between students and their personal academic tutors,^40,41^ rather than those ‘just’ delivering lectures and seminars. Walker and Gleaves identify elements of caring practice from the literature (p.65): ^39^

Caring teaching in practice appears to comprise two main pedagogic elements – the active fostering of and maintenance of pedagogic relationships above all else, and within these, the privileging of trust, acceptance, diligence and individual attentiveness. These pedagogic bonds hold at their centre notions of reciprocity, the situation of ‘the other’ and the significance of reflexivity in responding appropriately.

This is a strong account, undoubtedly influenced by a small sample of six, selected using reputational case selection where academics were nominated for inclusion because they were perceived as particularly caring by colleagues. The way in which reciprocity is manifest is not detailed and would seem challenging in what is surely an asymmetrical relationship. There appears to be a limited but growing literature on teaching as caring practice in higher education^42^ but it is nowhere near as extensive as in the nursing literature. For the purposes of this comparison, ask yourself about the relationships you have with students. Ask your colleagues. Are they caring relationships? I have in the past cautioned against getting too involved with student welfare, but I have heard colleagues say ‘well, I am a nurse’ as if this was by itself sufficient explanation and justification.

## Implications

It is the nature of this sort of wide-ranging narrative account that the analysis on each point is in need of more detail. The (loose) argument from analogy does not argue for equivalence, but I suggest that the analogy holds sufficiently that ‘nursing ethics’ ought to inform the relationships that nursing academics have with students. I say inform, as my modest intention is to provoke reflection in my fellow nurse academics not only about their own practice, but also about policies their employing institutions require them to follow. My conclusion is that some of what we do conflicts with nursing ethics and impedes the cultivation of character. This is clearly demonstrated in practices of control and surveillance.

## Control and coercion: Attendance and follow-up

NMC approved courses require a specific amount of time (2300 hours) in practice, and these are strictly measured.^43^ But this measurement is not a requirement for ‘theory’ hours which is assumed within course design.^44^ Detailed attendance monitoring and the subsequent chasing of non-attenders, and the accompanying disapproval, confuses teaching with learning. Do we assume that students cannot meet learning outcomes unless they do it in a manner of our choosing, that it is not their responsibility, as autonomous learners, to decide how best to learn? This is a paradigm case of disempowerment, standing in tension with student-centred learning and in complete opposition with analogous patient centred care. I do not suggest that universities use direct power to drag students kicking and screaming into the classroom, but various forms of coercive language and disapproval are employed and visible in university policies of the sort that are simply incompatible with nursing ethics. Another example, from the University of Exeter: ‘Students registered on University of Exeter programmes are required to…engage with and participate in all asynchronous learning and teaching activities, guided independent and group study, assessment and feedback…’^45^ Students are, of course, required to engage with assessment if they want academic credit, but to *require* engagement with all forms of learning activity is to enforce process and control in search of outcome. If our students treated their patients the way that universities treat their students, they would be liable to disciplinary action.

## Surveillance: big data and learner analytics

Pristilli (p.43) defines learner analytics and explains their purpose:^46^

learning analytics focuses on “the measurement, collection, analysis and reporting of data about learners and their contexts, for purposes of understanding and optimizing learning and the environments in which it occurs.” This is a prime example of institutions turning to business intelligence techniques that utilize prescriptive interventions to augment and support student success.

Increasingly, data analytics are used to monitor students’ activities, for example attendance and the amount of time spent on the university virtual learning environment (VLE). Students are not offered choice about whether their data are monitored and used in this way,^47^ there’s no consent for opt-in. There is little research on data analytics in the educational context though some studies seem to suggest that students are comfortable with the use of big data,^48^ and have high expectations of it,^49^ though students also appear to lack awareness of what data are collected and how they are being used.^50^ Foster and Siddle’s^51^ paper claimed that data analytics are effective in identifying students at risk of not progressing and collected data from log-ins to the VLE, VLE learning room usage, submissions of assignments, library book loans and card swipes into buildings on a daily basis. A guide to using data for personal tutoring produced by a leading commercial company is supported by 27 references, only 4 of which are from academic journals, the vast majority being blog posts.^52^ As far as nursing is concerned a theoretical paper by Jeffreys, from the US starts (p.181):^53^

Given the impact of the COVID-19 pandemic on every aspect of life, nursing programs and faculty will need to track, trend, and compare student data and outcomes via various trajectory pathways and consider the many variables influencing student retention, academic progression, and success in new and different ways.

The words ethics or ethical don’t appear in this paper. Jeffreys’ explanation, and the assumption that it is a ‘need’ is in tension with nursing ethics based on relationships, and there is a lack of clarity in the narrative about benefit. In whose interests are these coercive activities and intrusive surveillance performed? If it is students’ interest then we are being paternalistic, in direct opposition to respecting autonomy,^54^ one of the most fundamental principles in healthcare ethics and something that ethical nurses take great care in avoiding.^55^

Alternatively, if the beneficiary of these interventions is the university implementing them, they can be regarded as outright authoritarian, and, it must be remembered, this is in the context of students paying tuition fees and incurring large amounts of debt to do so. A paying customer is being coerced and monitored to benefit the merchant. In Foster and Siddle’s ^51^ paper the justification for choosing failure to obtain a first or upper-second class degree as an indicator of an ‘at risk’ student is that it ‘is often considered by students as a highly desirable outcome, often necessary for post graduate study or graduate-level employment’ (p.846). But this narrative fails to acknowledge university interests in increasing the proportion of high awards in a competitive environment of a ‘tyranny of metrics.’^56^ A claim that the interventions benefit students and universities simply acknowledges both paternalism and authoritarianism.

Research on identifying ‘at risk’ students does not extend to assessment of the interventions utilised. Foster and Siddle’s paper^51^ excludes this, and a case study^57^ at the University of Essex, available on a commercial website makes bold claims about reductions of withdrawal, failure or transfer without detailing the interventions used. A sceptic might also note the inclusion of transferring students in the figures; these students are not lost to education, only to a specific institution. The paper acknowledges the possibility of the influence on the metrics of a ‘no detriment’ approach to student support and success during the COVID pandemic.

## Attrition and the cultivation of character

Student nurse attrition is a ‘wicked’^58^ and complex problem complicated by the effects of the COVID pandemic. It is an international phenomenon^59^ much broader than university academic failure, including clinical challenges and unrealistic expectations, with the biggest reason in the UK being financial pressures.^60^ Any intervention that reduces attrition might be considered a good thing but the implication of coercive and surveillance policies and practices is that there will be a number of students who have arrived at the end of their course just because they have been coerced into the classroom. Coupled with concerns about failure to fail^61^ and academic grade inflation,^62^ it might be considered that even though metrics are improved, and student and university success is celebrated, that this system does not facilitate a process of character cultivation.

Character is cultivated and virtues acquired by repeated performance of acts, and it is difficult to see how this can be achieved when motivation for some students is at least partly the result of wishing to avoid censure from the university, albeit dressed up as concern. As Spieltheener^63^ pointed out, fear of punishment provides a very good reason for complying with a code of ethics, but a nurse doing this would not be considered virtuous or of good character. Behaviour is not internalised; it is compliance rather than adherence to an external standard, participation-in-name-only.^31^ Character cultivation in pre-registration nursing programmes requires consideration of both virtues of thought and virtues of character, and though this might also be said for all programmes in the academy, the combination has special significance in vocational higher education. There is a significant literature on virtue ethics in nursing but less so on acquisition of the intellectual virtues. This paper simply notes that the acquisition of intellectual virtue requires both motivation and skill^64^ just as a virtuous person ‘deliberates toward morally good ends and acts with the *intention* to achieve those ends’ (p. 85, emphasis in original).^65^ This paper does not add to the literature by examining how the intellectual virtues can be cultivated, it simply suggests that motivation and intention to become a virtuous nurse are unlikely to be cultivated in an environment which sees success not in terms of cultivating ethically aware practitioners, but instead values outcomes and statistics on progression, degree classification and satisfaction and is prepared to use coercion and surveillance in order to obtain them. Nurses should not treat their patients like this, and nor should we nurse academics treat our students like it.

## Conclusion

The intention of this paper is modest. I simply ask nurse academics to consider whether their practices, and those of their employers are consistent with their conception of nursing ethics. I have concluded that some are not. It is not intended to be a detailed ethical analysis of the practices that I have critiqued, though this will surely be needed as policies and practices in nursing education develop. More widely than these policies, cultivating character and virtue in nursing is becoming more challenging as cohort numbers rise^66^ and pressure for success intensifies. In the UK, the NHS is perilously short of nurses, and some universities are perilously short of money.^67^ However, these pressures do not justify unethical treatment of students, and nurse lecturers and more especially their managers would do well to return to the professional ethics of nursing to question and guide their practice.

## Note

1. In the UK there is no lower age limit but nearly all student nurses are over 18.
